# Biocontrol of the causal brown patch pathogen *Rhizoctonia solani* by *Bacillus velezensis* GH1-13 and development of a bacterial strain specific detection method

**DOI:** 10.3389/fpls.2022.1091030

**Published:** 2023-01-09

**Authors:** Gahee Lee, Hyeongju Choi, Haifeng Liu, Yun-Hyeong Han, Narayan Chandra Paul, Gui Hwan Han, Hyunsook Kim, Pyoung Il Kim, Sun-Il Seo, Jaekyeong Song, Hyunkyu Sang

**Affiliations:** ^1^ Department of Integrative Food, Bioscience and Biotechnology, Chonnam National University, Gwangju, Republic of Korea; ^2^ Division of Food and Biotechnology, Chonnam National University, Gwangju, Republic of Korea; ^3^ Damyang-gun Agricultural Technology Center, Damyang, Republic of Korea; ^4^ Kumho Life Science Laboratory, Chonnam National University, Gwangju, Republic of Korea; ^5^ Center for Industrialization of Agricultural and Livestock Microorganisms, Jeongeup, Republic of Korea; ^6^ Boran Pharma, Seoul, Republic of Korea; ^7^ Agricultural Microbiology Division, National Institute of Agricultural Sciences, Rural Development Administration, Wanju, Republic of Korea

**Keywords:** *Bacillus velezensis*, biocontrol, brown patch, pan-genome, detection, *Rhizoctonia solani*

## Abstract

Brown patch caused by the basidiomycete fungus *Rhizoctonia solani* is an economically important disease of cool-season turfgrasses. In order to manage the disease, different types of fungicides have been applied, but the negative impact of fungicides on the environment continues to rise. In this study, the beneficial bacteria *Bacillus velezensis* GH1-13 was characterized as a potential biocontrol agent to manage brown patch disease. The strain GH1-13 strongly inhibited the mycelial growth of turf pathogens including different anastomosis groups of *R. solani* causing brown patch and large patch. *R. solani* AG2-2(IIIB) hyphae were morphologically changed, and fungal cell death resulted from exposure to the strain GH1-13. In addition, the compatibility of fungicides with the bacterial strain, and the combined application of fungicide azoxystrobin and the strain in brown patch control on creeping bentgrass indicated that the strain could serve as a biocontrol agent. To develop strain-specific detection method, two unique genes from chromosome and plasmid of GH1-13 were found using pan-genome analysis of 364 *Bacillus* strains. The unique gene from chromosome was successfully detected using both SYBR Green and TaqMan qPCR methods in bacterial DNA or soil DNA samples. This study suggests that application of GH1-13 offers an environmentally friendly approach *via* reducing fungicide application rates. Furthermore, the developed pipeline of strain-specific detection method could be a useful tool for detecting and studying the dynamics of specific biocontrol agents.

## 1 Introduction

Brown patch caused by the basidiomycete fungus *Rhizoctonia solani* Kühn is a disease of cool-season grasses including, annual bluegrass, creeping bentgrass, and tall fescue. The disease occurs in warm and humid weather and is more prevalent with prolonged leaf wetness and high temperature. The symptoms of the disease appear roughly circular patches with brown, tan, or yellow color. (shown in https://www.turffiles.ncsu.edu/diseases-in-turf/brown-patch-in-turf/). In addition to brown patch, this soilborne pathogen causes diseases on a wide range of hosts including, economically important crops such as soybean, rice, and potatoes (Lee and Rush, 1983; Woodhall et al., 2007; Ajayi-Oyetunde and Bradley, 2017). Species of *R. solani* are classified into 14 different anastomosis groups (AGs) based on the ability of their hyphae to fuse with one another (Ajayi-Oyetunde and Bradley, 2018). Among them, six AGs (AG 1, AG 2-2, AG 3, AG 5, AG 5, and AG 6) have been isolated from cool-season turfgrasses (Koehler and Shew, 2017), and AG 1-IB, and AG 2-2-IIIB predominantly cause brown patch on the turfgrasses (Zhang and Dernoeden, 1995).

To reduce brown patch, the following cultural practices have been recommended: proper irrigation and early morning mowing to minimize the duration of leaf wetness, removing surrounding trees and shrubs to facilitate water evaporation and improve soil drainage, and application of proper amounts of nitrogen fertilizer (Tredway and Burpee, 2001). However, these cultural practices alone are insufficient to control brown patch when the disease is severe. Fungicides have been mainly applied to golf courses mainly to manage brown patch. The classes of fungicides which have been used are benzimidazole, carbamates, dicarboximide, demethylation inhibitors (DMIs), phenylpyrroles, quinone outside inhibitors (QoIs), succinate dehydrogenase inhibitors (SDHIs), and some multisite contact fungicides. These are applied preventatively and/or curatively during summer months to manage brown patch (Tredway and Burpee, 2001, shown in https://extension.psu.edu/turfgrass-diseases-brown-patch-causal-fungus-rhizoctonia-solani). Applications of fungicides control fungal diseases most effectively but the repeated and prolonged use of fungicides has negative impacts on environments and can develop fungicide-resistant populations. To manage diseases in an environmentally friendly and sustainable manner, biological controls have been suggested as alternative methods to fungicide applications. This approach uses microorganism(s) to manage plant disease by reducing pathogen activities and inducing plant defense mechanisms (Nelson et al., 1994). Various microorganisms such as *Paenibacillus ehimensis*, *Stenotrophomonas maltophilia*, and *Trichoderma harzianum* have been characterized for controlling brown patch (Yuen and Zhang, 2001; Guerrero et al., 2008; Jeon et al., 2015), but most commercially available biological products (active ingredients) for brown patch management are *Bacillus subtilis* (e.g. strain QST713, Rhapsody from Bayer; strain GB03, Companion from Growth Products Ltd).

Since some *Bacillus* species have been known to possess strong antagonistic activity against plant pathogens and promote plant growth, more than 18 commercial *Bacillus* species-based products have been distributed worldwide (Fira et al., 2018). Among them, *B. velezensis* is a newly introduced species that has shown high antifungal activity against diverse phytopathogenic fungi and excellent disease control for different crops. For example, *B. velezensis* OEE1 isolated from the roots of olive trees displayed strong antifungal activity against *Verticillium dahliae*, the causal pathogen of Verticillium wilt of olive, and has been effective in controlling this disease of olive trees in the field (Azabou et al., 2020). Jiang et al. (2018) screened 100 bacterial strains against a gray mold pathogen *Botrytis cinerea* by *in vitro* dual culture assays and two *B. velezensis* strains showing the best antagonistic activity were selected. These strains controlled the disease on pepper plants by suppressing the growth and spore germination of the pathogen and inducing plant basal immunity and hydrogen peroxide accumulation in pepper. Some strains of *B. velezensis* also produced antimicrobial compounds such as lantibiotic ericin (Palazzini et al., 2016) and lipopeptides including fengycin, iturin, and surfactin (Cao et al., 2018) which can inhibit fungal and bacterial growth. It was also found that root colonization by *B. velezensis* FZB42 (formerly *B. amyloliquefaciens*) could elicit induced systemic resistance (ISR) and limit the reopening of stomata mediated by *Phytophthora nicotianae* in *Nicotiana benthamiana*, thus blocking the penetration of the host by the pathogen (Wu et al., 2018). Although these excellent properties of *B. velezensis* act as a biocontrol agent, this species has neither been characterized nor applied to control brown patch.

To protect against plant diseases, it is important to detect and identify plant pathogens. Many powerful methods have been developed and applied for target organism detection, such as enzyme-linked immunosorbent assay (ELISA), loop-mediated isothermal amplification (LAMP), and nucleic acid amplification-based methods including polymerase chain reaction (PCR) and quantitative PCR (qPCR) (Notomi, 2000; Cho and Irudayaraj, 2013; Martinelli et al., 2015). These methods have been applied for the detection and quantification of biocontrol agents (Savazzini et al., 2008; Gonçalves et al., 2014; Sanzani et al., 2014; Rotolo et al., 2016). However, there are limitations that the markers used for detection may not be strain-specific and can be detected in other strains of the same species or related and unrelated organisms. To date, there are more than 350,000 genomes of prokaryotes and 18,000 genomes of eukaryotes deposited in NCBI (https://www.ncbi.nlm.nih.gov/genome/browse#!/overview/). Using these publicly available genomic data and pan-genome analysis (Tettelin et al., 2005; Chaudhari et al., 2016), the unique genes of a single strain can be mined, and the genes can be utilized as markers for strain-specific detection of a biocontrol agent.

*B. velezensis* GH1-13 isolated from a paddy field displayed strong antimicrobial activity against rice and other crop diseases (Kim et al., 2016). This strain can be a good candidate as a biological agent to control turf diseases including, brown patch. In addition, using the genome sequences of this strain (Kim et al., 2017) unique genes of GH1-13 can be found with other genomic data available in NCBI. Our study aimed to (i) characterize the *B. velezensis* GH1-13 as a biocontrol agent against brown patch pathogens and determine the efficacy of *B. velezensis* GH1-13 either alone or in combination with a commercial fungicide to control brown patch; (ii) to develop a GH1-13 strain-specific detection method using the pan-genome and qRT-PCR analysis for studying colonization and dynamics of the bacterial strain in turfgrasses.

## 2 Materials and methods

### 2.1 Bacterial, fungal, and oomycete strains and fungicides

A *B. velezensis* strain GH1-13 was isolated from a reclaimed paddy field on Wando Island in Korea (Kim et al., 2016). To investigate its antifungal activities against turf pathogens, fungal and oomycete strains causing or potentially causing brown patch [*R. solani* AG-1(IA) (KACC 40103), *R. solani* AG-1(IB) (KACC 40109), *R. solani* AG-4 (KACC 40143), *R. solani* AG2-2(IIIB) (KACC 40151)], large patch [*R. solani* AG2-2(IV) (KACC 40132)], yellow patch [*R. cerealis* (KACC 40154)], Pythium blight [*Pythium ultimum* (KACC 40705)] were obtained from the Korean Agricultural Culture Collection (KACC), National Institute of Agricultural Sciences, Rural Development Administration, Jeonju, Korea. In addition, one strain (CMML20-28) of the dollar spot pathogen *Clarireedia jacksonii* isolated from creeping bentgrass leaf blade and identified by multiple markers (unpublished data) was also used in this study. This bacterial strain was cultured in tryptic soy broth (TSB, Difco, Sparks, MD, USA) and tryptic soy agar (TSA), and the fungal and oomycete strains were cultured on potato dextrose agar (PDA, Difco, Sparks, MD, USA). The cultured bacterial, fungal and oomycete strains were maintained in 20% glycerol stock solution at − 80°C and on TSA and PDA plates at 4 °C prior to use.

Two fungicides, azoxystrobin (Otiva, Syngenta, containing 21.7% of active ingredient) and fluxapyroxad (Cardis, Nonghyup chemical, containing 15.3% of active ingredient), were used in the study. Stock solutions (10,000 μg ml^-1^) of azoxystrobin and fluxapyroxad were prepared and stored at 4°C before use.

### 2.2 Dual culture assay

Antifungal activity of *B. velezensis* GH1-13 against stains of five different anastomosis groups [AG-1(IA), AG-1(IB), AG-4, AG2-2(IIIB), and AG2-2(IV)] of *R. solani*, *R. cerealis*, *Pythium ultimum*, and *C. jacksonii* was tested to investigate the potential for the bacterium to control turf pathogens. At first, a single colony of *B. velezensis* GH1-13 was inoculated and grown in 5 ml of TSB overnight at 30°C at 150 rpm in a shaking incubator. Then, 150 μl of the bacterial culture was transferred to 5 ml new TSB media. After growing the bacterial culture for 6 h, 10 μl of the culture (optical density (OD) value = 0.4; 1×10^7^cfu ml^-1^) was inoculated on three positions 20 to 25 mm from the midpoint where the fungal agar plug was to be placed. Eight strains were grown on PDA for 4-5 days at 25°C, and one 5mm agar plug taken from the edge of the actively growing colonies of each isolate was placed at the center of each of the three replicate PDA plates of each isolate one day after the bacterial inoculation. Pathogens without bacteria served as the control. All plates were incubated at 25°C in darkness. The colony diameter of each pathogen in the absence or presence of GH1-13 was observed and was measured 2-14 days after inoculation. The inhibition rate was calculated using the following formula (Ji et al., 2013):


$$Inhibition\;rate\;\left(\%\right)\;=\frac{A1-\;A2}{A1}\;\times \;100$$

A1 is the average diameter of colonies from each pathogen and A2 is the average diameter of colonies in the presence of GH1-13. The experiment was performed twice with three replicates.

### 2.3 Microscopic analysis

To evaluate the antifungal efficacy of *B. velezensis* GH1-13 against brown pathogen, the strain KACC 40151 of *R. solani* AG2-2(IIIB) was co-cultivated with the strain GH1-13 on PDA media at 25°C for 8 days. Mycelia of *R. solani* AG2-2(IIIB) that were adjacent to the bacterium or growing on the opposite sides from the bacterium were placed in 10 μl of neutral red (0.1 μg ml^-1^, DaeJung) or Evans blue (0.5 μg ml^-1^, Alfa Aesar) on separate glass slides. The slides were incubated for 5 min at room temperature and washed 3 times with sterilized ddH_2_O. The stained mycelia and the morphology of mycelia were observed, and microscopic images of mycelia were photographed under a light microscope (Olympus, Tokyo, Japan). The experiments were conducted twice, and more than 50 images of mycelia per treatment were taken.

### 2.4 Compatibility of fungicides with *B. velezensis* GH1-13

Compatibility tests of fungicides with GH1-13 were conducted to investigate the effect of fungicides commonly used in turfgrass on *B. velezensis* GH1-13. The strain GH1-13 was grown in nutrient broth (NB, Difco, Sparks, MD, USA) medium on a shaking incubator at 180 rpm at 28°C for 24 hrs. Then, 150 μl of bacterial culture was added to the flasks containing 20 ml of NB medium with and without fungicides (20 and 100 μg ml^-1^ of azoxystrobin and fluxapyroxad). After incubation at 180 rpm and 28°C for 24 hrs, one ml of the culture from each flask was serially diluted at 10-fold intervals in NB, and the 10^-4^ fold-diluted culture was plated onto nutrient agar (NA) medium. The number of individual bacterial colonies on each plate was counted after being cultured for 24 hrs at 28°C. The experiment was conducted three times with three replications.

### 2.5 *In planta* antifungal assay

The efficacy of *B. velezensis* GH1-13 and fungicide (azoxystrobin) against the brown patch pathogen was tested on creeping bentgrass. The bentgrass was grown in vinyl pots containing 120g soil in a growth room at 23 ± 5 ˚C for 3 weeks. The strain *R. solani* AG2-2 (IIIB) KACC 40151 was grown in PDB for 3-4 days, and mycelia were ground for inoculation. Four different treatments were prepared as follows: GH1-13 suspension (1×10^6^cfu ml^-1^), azoxystrobin (20 μg ml^-1^), GH1-13 suspension (1×10^6^cfu ml^-1^) + 50 percentage of azoxystrobin (10 μg ml^-1^), and 50 percentage of azoxystrobin (10 μg ml^-1^). Sterilized water was used as an untreated control. After applications of the four treatments, the ground mycelia were inoculated on bentgrass. Disease severity (using a 0 to 7 scale; 0 = healthy plant, 1 = less than 15% infection, 2 = 15-30% infection, 3 = 30-45% infection, 4 = 45-60% infection, 5 = 60-75% infection, 6 = 75-90% infection, 7 = dead plant) was rated 14 days after inoculation. The experiment was conducted twice with three replicates. The disease severity index (%) was calculated (Chiang et al., 2017) as follows:


$$DSI\;\left(\%\right)=\frac{\text{Σ}\left(n\times disease\;severity\;scale\right)}{N\times the\;highest\;scale}\;\times 100$$

Here, DSI indicates disease severity index, n = the number of turfgrass pots in each scale, and N = total number of pots tested.

### 2.6 Pan-genome analysis

Pan-genome analysis was conducted by the BPGA program (Chaudhari et al., 2016; Kim et al., 2017) to illustrate the genomic features specific to strain GH1-13. A total of 364 genome sequences of *Bacillus* strains were derived from the nucleotide database of NCBI. Among them, 269 genome sequences were from *B*. *velezensis*; 95 from other species of *Bacillus*. All genes of these strains were analyzed by the NJ method in the pan-genome analysis pipeline with a 50% cut-off for protein sequence identity. Orthologs protein coding sequences were used to identify core conserved genes and strain-specific genes.

### 2.7 Strain GH1-13 specific detection by SYBR Green qPCR and TaqMan qPCR

To detect the strain GH1-13 specifically, two unique genes from plasmid and chromosomal DNA sequences of GH1-13 were selected and used for SYBR Green qPCR and TaqMan qPCR analysis. RNA of GH1-13 was extracted using an RNAiso plus kit (Takara, Shiga, Japan), and reverse transcription of RNA into cDNA was conducted by a PrimeScript 1st Strand cDNA synthesis kit (Takara, Shiga, Japan). For specific amplification of the plasmid DNA, specific primers (F_uniqueP/R_uniqueP) were designed and analyzed for specificity by BLAST. For amplification of the unique gene from chromosomal DNA, specific primers (F_uniqueC/R_uniqueC and Ft_uniqueC/Rt_uniqueC) and a TaqMan probe were designed. The sequences of primers and probes are listed in **Table 1**. To test the specificity of qPCR, genomic DNA of different species (strains) including *B. velezensis* strains (GH1-13, JC-14 and TSA34-9), *B*. *subtilis* JC-15, *Escherichia coli*, and *R. solani* were used with the same DNA concentration of 0.1 ng/µl. For the qPCR sensitivity assay, DNA of GH1-13 was tested with concentrations ranging from 1,000,000 fg/µl (1ng) to 10 fg/µl (10-fold serial dilutions). SYBR Green qPCR analysis was performed using Universal SYBR Green Supermix (Bio-Rad, California, USA). The reaction mixtures contained 1 µl of each primer and 10 µl of SYBR Green Supermix, 1µl of template DNA, and 7 µl of sterile water. The TaqMan qPCR reaction mixtures contained 1 µl of each primer and 10 µl of Universal Probes Supermix (Bio-Rad, California, USA), 1 µl of template DNA, 1 µl of the probe, and 6 µl of sterile water.

**Table 1:** Primer and probes used in the present study.

Primer/probe	Sequence (5’ to 3’)	Length (base pairs)	Temperature (°C)
F_uniqueC	GGGAATTTCAGATTACTGCGA	21	60
R_uniqueC	GACTCTTCATTCGAAATTAACAAGA	25	60
Ft_uniqueC	ACTACATATCCAAACAGATTT	21	55
Probe^a^	TGGGTTTCGCAGTAATCTGAAATTCCC	27	67
Rt_uniqueC	TCCCTGTTCTTCATCTTC	18	56
F_uniqueP	ATGGAATACATCTTGAAAGCTCTG	24	61
R_uniqueP	GACTTGCCAAGGGTACAAATTC	22	62

a Probe contains 5’ fluorophores ‘‘6-FAM’’ and 3’ quenchers ‘‘BHQ-1”.

### 2.8 Detection of GH1-13 from rhizosphere of creeping bentgrass in the pots

To detect the strain GH1-13 from the rhizosphere, 1-week old creeping bentgrass grown in pots were used for three treatments (GH1-13 treated, TSA34-9 treated, and non-inoculated) with three pot replicates of each treatment. In the bacterial treatments, 5 ml suspension (1×10^6^cfu ml^-1^) was inoculated twice into the pots, with an interval of three days. The bentgrass pots were placed in a greenhouse under at 25°C during the assay. After three days of cultivation, approximately 500 mg of rhizosphere soil was sampled from each pot, and genomic DNA of the soil samples was extracted using a FastDNA Spin Kit for Soil (MP Bio, Santa Ana, USA). SYBR Green and TaqMan qPCR analyses were conducted to detect the unique gene of GH1-13 from chromosomal DNA of soil samples using the primer pairs F_uniqueC/R_uniqueC and Ft_uniqueC/Rt_uniqueC.

### 2.9 Detection of GH1-13 from rhizosphere of creeping bentgrass in the field

To detect the strain GH1-13 in the field, 250 ml of bacteria suspension (1×10^6^cfu ml^-1^) was treated on creeping bentgrass in Chonnam National University experimental field in Naju, South Korea, with three replications in October, 2021. Control plots were treated with the same volume of distilled water. Creeping bentgrass rhizosphere soil samples (500 mg) were randomly collected from each treatment after 3 h, 6 months and 9 months after the treatment. The method of DNA extraction from the soil samples was same as described previously. For specific detection of GH1-13 chromosomal unique gene, SYBR Green qPCR analysis was performed with three replications using primer pair F_uniqueC/R_uniqueC.

### 2.10 Field efficacy of GH1-13 for management of brown patch

Field efficacy test of GH1-13 for management of brown patch was conducted on the creeping bentgrass in the Chonnam National University experimental fields in Naju, South Korea in 2021 and 2022. The design and size of the experimental plots were followed the methods described by Sang et al. (2019). A randomized complete block design (RCBD) was used with three replications. Each plot was 40.0 × 60.0 cm with 3.0-cm buffer strips between each plot. Four different treatments were applied as follows (GH1-13 suspension (1×10^6^cfu ml^-1^), azoxystrobin (20 μg ml^-1^), GH1-13 suspension (1×10^6^cfu ml^-1^) + 50 percentage of azoxystrobin (10 μg ml^-1^), and 50 percentage of azoxystrobin [10 μg ml^-1^)] and distilled water served as control. The strain *R. solani* AG2-2 (IIIB) KACC 40151 was also inoculated a day after the treatment of GH1-13. The biocontrol agent GH1-13 and the pathogen KACC 40151 applied three times with two weeks interval from the first application. Disease severity was calculated 2 weeks after the last treatment using 0 to 10 scale with the infection percentage of 0 = healthy plant, 1= less than 10% infection, 2 = 11-20%, 3 = 21-30%, 4 = 31-40%, 5 = 41-50%, 6 = 51-60%, 7 = 61-70%, 8 = 71-80%, 9 = 81-90%, 10 = 91-100% or dead plant. The disease severity index (%) was calculated using the aforementioned methods.

### 2.11 Statistical analysis

All statistical analysis was performed using ‘JMP Statistical Discovery’ software package, version 10.0 (SAS institute). One-way ANOVA was used to assess the analysis of datasets from dual culture assay, compatibility of fungicides, pot, and field assay. Fisher’s least significant difference (LSD) means comparison (P = 0.05) was performed to determine statistical significance. The statistical analysis of association between variables was compared using the pairwise Pearson’s correlation coefficient.

## 3 Results

### 3.1 Antifungal activity of GH1-13 against turf pathogens

**Figure 1:**
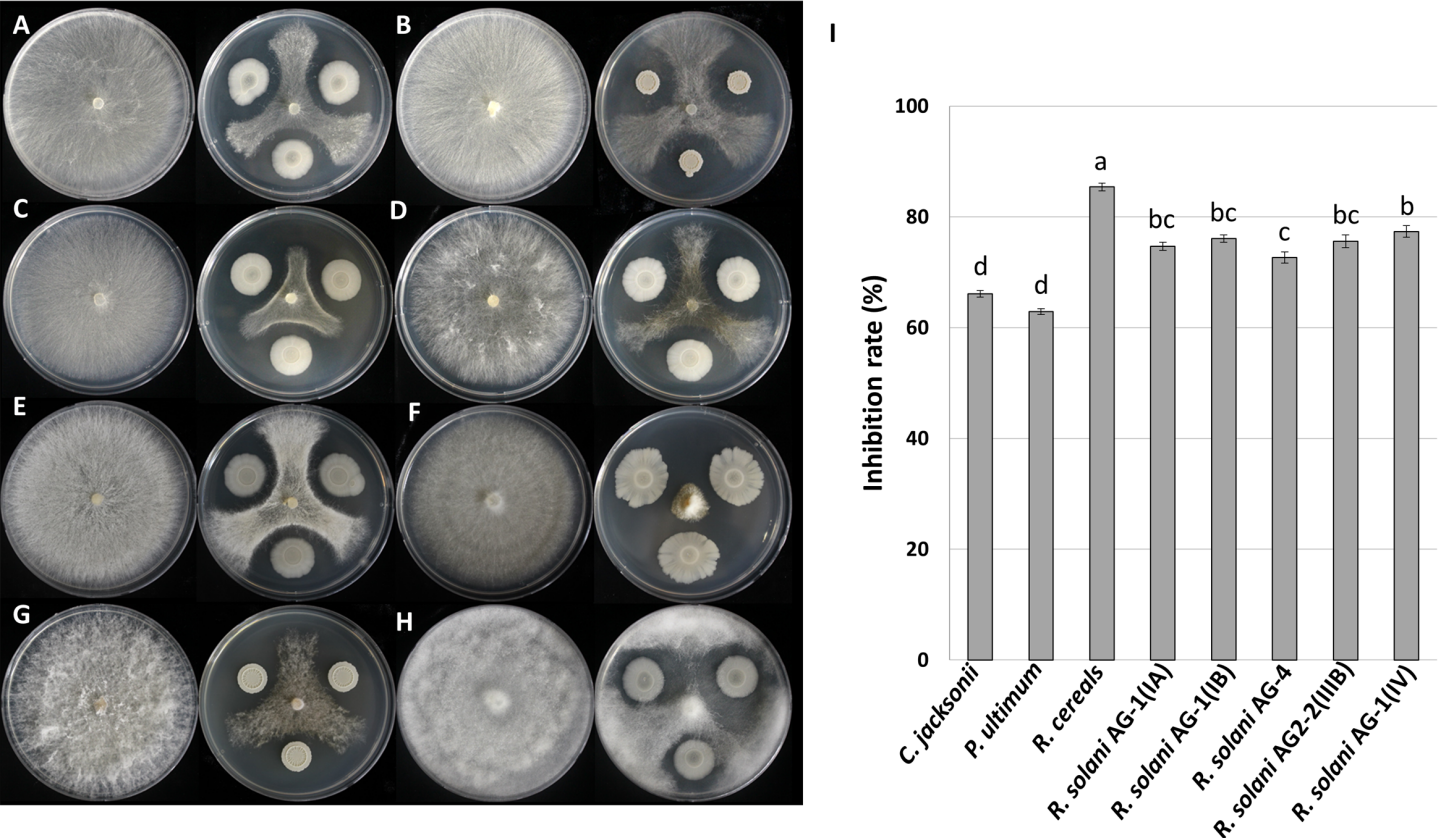
Antifungal activity of *Bacillus velezensis* GH1-13 against eight plant pathogens. **(A)** *Rhizoctonia solani* AG-1 (IA), **(B)** *R. solani* AG-1 (IB), **(C)** *R. solani* AG2-2 (IIIB), **(D)** *R. solani* AG2-2 (IV), **(E)** *R. solani* AG-4, **(F)** *Rhizoctonia cerealis*, **(G)** *Clarireedia jacksonii*, and **(H)** *Pythium ultimum*, and **(I)** inhibition rates of the pathogens. The bars followed by the same letter are not significantly different at P< 0.05 in Tukey’s HSD test.

To assess the inhibitory ability of *B. velezensis* GH1-13 against turf pathogens, *in vitro* dual culture assays against eight fungal and oomycete strains were conducted. Strain GH1-13 demonstrated strong inhibitory activity against the mycelial growth of tested pathogenic strains. This dual culture assay allowed the relative growth of pathogenic fungal and oomycete mycelia to be quantitatively assessed. Considerable variability was observed in the inhibitory activity against all eight pathogens. Strain GH1-13 showed the best inhibitory activity against *R. cerealis*, reducing fungal growth by 85%. Against the other 7 strains, the bacterium GH1-13 showed inhibition in the range of 63-77%. The *R. solani* strains causing brown patch or large patch were inhibited by GH1-13 in a range of 70-77%, a significantly higher inhibition rate than against the strains *C. jacksonii* and *P. ultimum* (**Figure 1**). These results indicate that the GH1-13 strain is a good candidate as a potential biocomponent for turfgrass fungal and oomycete pathogens.

### 3.2 Morphological changes of *Rhizoctonia solani* by treatment of GH1-13

**Figure 2:**
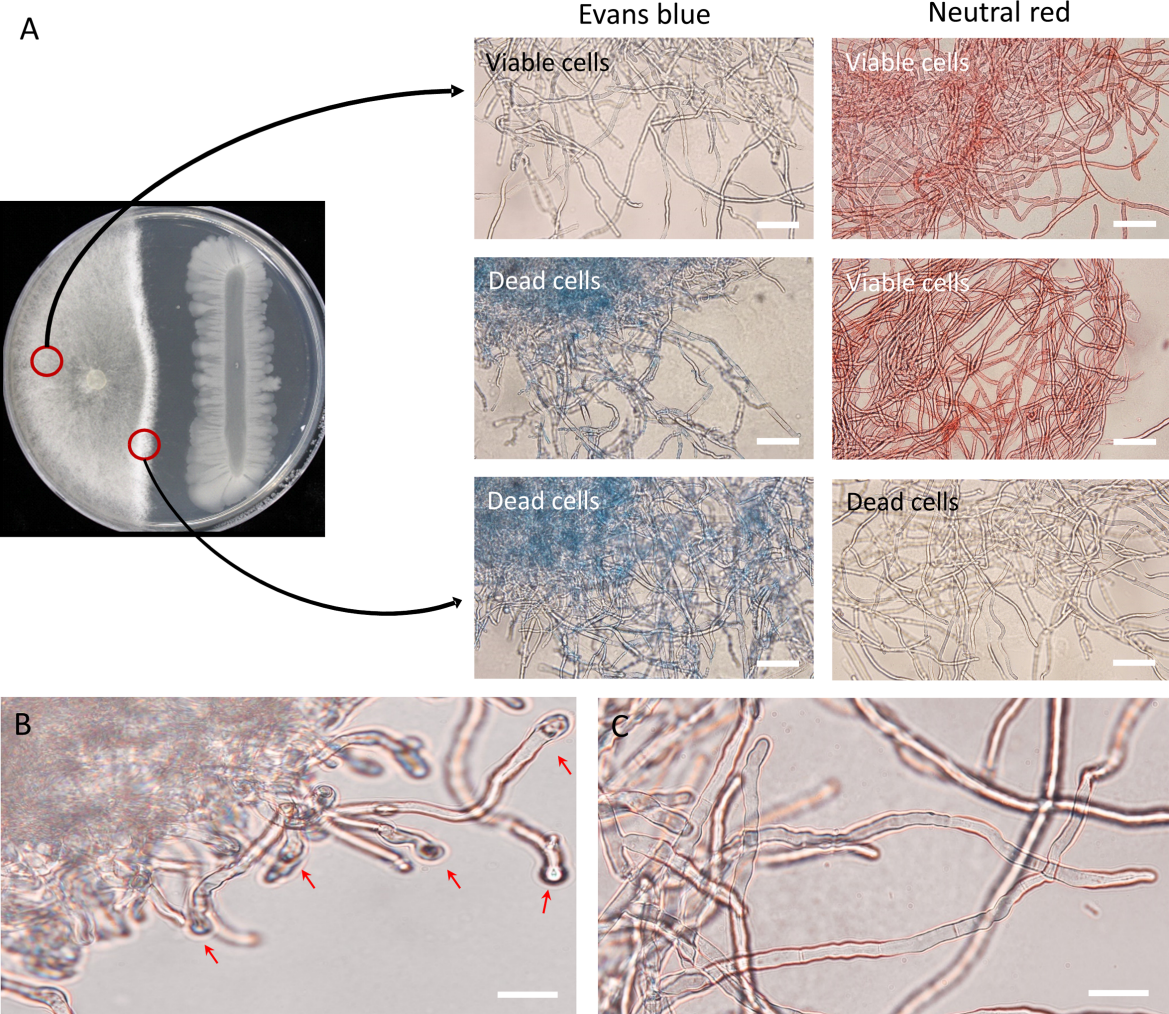
Morphological observation of inhibition effect of *Bacillus velezensis* GH1-13 against *R. solani* AG2-2 (IIIB). **(A)** Mycelia of *R. solani* were stained with Evans blue and neutral red after co-cultivation for 8 days; mycelia growing near GH1-13 stained blue, while those in the control zone exhibited only normal color; in contrast, mycelia that were co-cultured with GH1-13 did not stain red. **(B)** Roundish cells were observed at terminal hyphae of *R. solani* during dual culture. **(C)** Mycelia in the control region showed no inhibition. Scale bars = 50 μm.

Mycelia of *R*. *solani* were observed under the microscope after Evans blue and neutral red staining to investigate the viability and morphological changes of *R. solani* AG2-2(IIIB) by the *B. velezensis* GH1-13. In Evans blue stains, dead cells become blue, and in neutral red stains, viable or living cells are colored red. After 8 days of co-cultivation of the pathogen and the antagonistic bacteria, dramatic breakage of the mycelia of *R*. *solani* near the GH1-13 contact zone were observed and mycelia were stained blue, while the mycelia with no alteration in the cell membrane maintained their natural coloration or faint blue. In contrast, mycelia that were adjacent to GH1-13 did not stain red (consistent with dead cells), and the viable cells were observed to be red (**Figure 2A**). In the GH1-13 growth-inhibited mycelia of *R*. *solani*, distinct globoid cells were observed at the terminal hyphae (**Figure 2B**), while the control hyphal cells without inhibition were usually linear (**Figure 2C**).

### 3.3 Effect of fungicides on GH1-13

**Figure 3:**
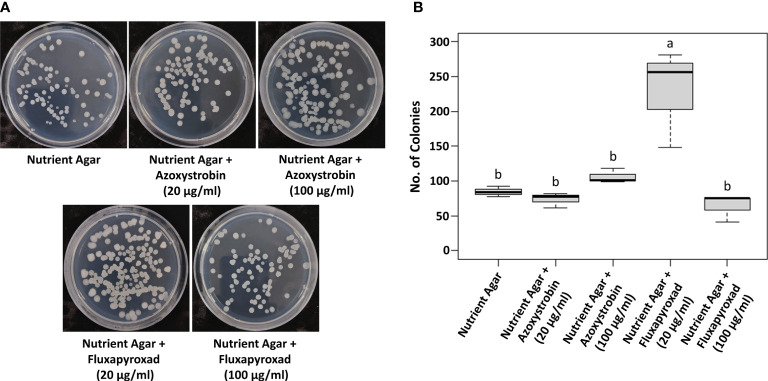
Compatibility of fungicides, azoxystrobin and fluxapyroxad, with *Bacillus velezensis* GH1-13. **(A)** Bacterial colonies on NA medium (control) and NA medium amended with 20 μg ml^-1^ and 100 μg ml^-1^ of azoxystrobin and fluxapyroxad. **(B)** Numbers of colonies were shown in the bar graph. Means followed by the same letter are not significantly different at P< 0.05 according to the Tukey’s HSD test.

It is essential to test the compatibility of *B. velezensis* GH1-13 with commonly used fungicides for their successful integration for disease inhibition. GH1-13 was compatible with concentrations of 20 and 100 μg ml^-1^ of azoxystrobin and fluxapyroxad, respectively (**Figure 3A**). The number of colonies of GH1-13 on 20 and 100 μg ml^-1^ of azoxystrobin and 100 μg ml^-1^ of fluxapyroxad amended NA medium was not significantly different from the number on non-amended NA medium. Interestingly, the low concentration (20 μg ml^-1^) of fluxapyroxad increased the number of colonies of GH1-13 significantly compared to other treatments or no treatment (**Figure 3B**).

### 3.4 Brown patch control by GH1-13 and fungicide azoxystrobin

**Figure 4:**
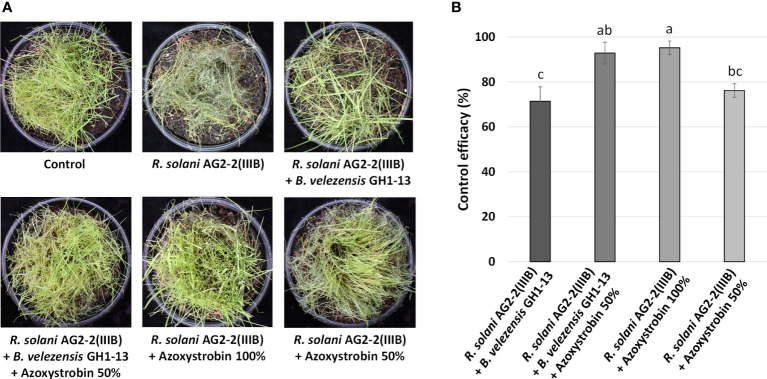
Effects of *Bacillus velezensis* GH1-13 and azoxystrobin on control of brown patch of creeping bentgrass in pot assay. **(A)** The symptoms of brown patch caused by *Rhizoctonia solani* AG2-2(IIIB) on creeping bentgrass and brown patch control by application of *B. velezensis* GH1-13 (1×10^6^cfu/ml), 50% azoxystrobin (10 μg ml^-1^), 100% azoxystrobin (20 μg ml^-1^), or the mixture of *B. velezensis* GH1-13 (1×10^6^cfu/ml) and 50% azoxystrobin (10 μg ml^-1^). **(B)** Control efficacy of different applications against brown patch. The bars followed by the same letter are not significantly different at P< 0.05 in Tukey’s HSD test.

The *B. velezensis* GH1-13 strain and fungicide azoxystrobin were used to control brown patch on creeping bentgrass. The control efficacy (%) of GH1-13 (1×10^6^cfu/ml) alone was 71.43± 6.3 and was similar to the efficacy (%) of 50% azoxystrobin (76.19 ± 3.0). The combined application of GH1-13 (1×10^6^cfu/ml) and 50% azoxystrobin (control efficacy: 92.86 ± 4.9) showed greater significant disease reduction than GH1-13 alone or 50% azoxystrobin and control efficacy similar to 100% azoxystrobin (95.24 ± 3.0) (**Figure 4**).

### 3.5 Pan-genome analysis

**Figure 5:**
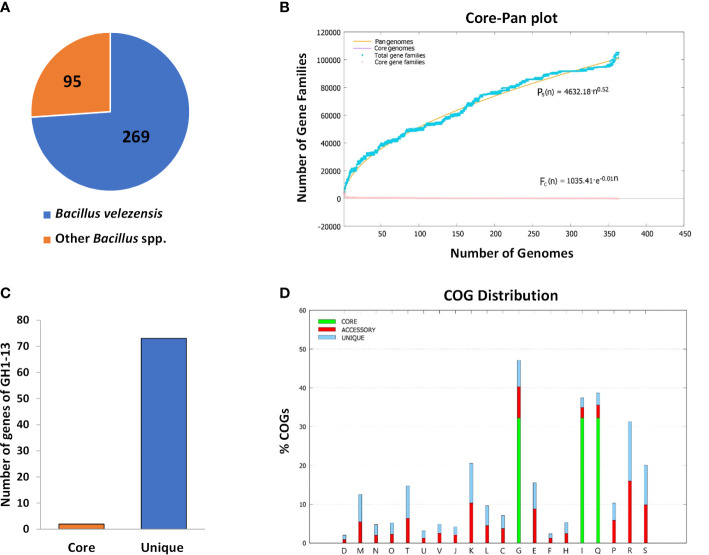
Pan-genome analysis of 364 strains within the *Bacillus* genus. **(A)** Pie chart displaying 269 strains of *B. velezensis* and other 95 *Bacillus* species. **(B)** 2 core genes and 73 unique genes were found in the strain GH1-13. **(C)** Mathematical modeling of pan-genomes and core genomes of *Bacillus* strains. **(D)** Detailed distribution of cluster of orthologous groups (COG) in core, accessory and unique genome.

The BPGA program was used to perform pipeline pan-genome analysis and all orthologous groups among the *Bacillus* strains tested were identified. In the analysis, core genes are a set of genes shared with all the tested strains. Unique genes represent the set of genes in each strain not shared with other strains. The results showed that the 364 genomes contained 2 core genes, and 73 genes were uniquely found in strain GH1-13 (**Figure 5A, B**). The pan-genome and core genome were curve fitted by an exponential decay model (**Figure 5C**). The predicted core, unique and accessory genes were further classified according to COG category, and their percentage in different functional categories was determined (**Figure 5D**). The Blastx tool was used to find potential matches and the function of those 73 unique genes of GH1-13. Among them, two unique genes (encoding hypothetical proteins) showing the most specific matches to GH1-13 were selected for further study. Of these two candidates, one (positioned at CP019040.1:1872828-1873025) originated from the chromosome and the other (positioned at CP019039.1:3715-3900) is from the plasmid in GH1-13 (BioProject: PRJNA359634). These two unique genes were thus used for further detection of strain GH1-13.

### 3.6 Strain GH1-13 specific detection by SYBR Green and TaqMan qPCR analysis

**Figure 6:**
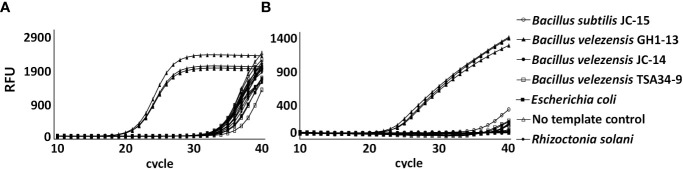
Amplification of chromosomal unique gene region of *Bacillus velezensis* GH1-13 from 0.1 ng initial DNA of *B. velezensis* GH1-13, *B. velezensis* JC-14, *B. velezensis* TSA34-9, *Bacillus subtilis* JC-15, *Escherichia coli*, *Rhizoctonia solani* and no template control by SYBR Green qPCR analysis using a primer pair F_uniqueC and R_uniqueC **(A)** and TaqMan qPCR analysis using a primer pair Ft_uniqueC and Rt_uniqueC **(B)**.

To detect the GH1-13 specifically, SYBR Green and TaqMan qPCR analyses using primer sets targeting the two unique genes were conducted with bacterial and fungal DNA samples. In a specificity assay of the chromosomal unique gene, DNA of the strain GH1-13 was specifically amplified by SYBR Green (**Figure 6A**) and TaqMan (**Figure 6B**) qPCR analysis and the assay could distinguish GH1-13 from the strains (JC-14, TSA34-9) of *B. velezensis* and other bacterial and fungal strains (*B*. *subtilis*, *Escherichia coli* and *R. solani*). Because the amplicon (75 bp) from TaqMan qPCR was different from the one in SYBR Green qPCR (81 bp), primer pair (Ft_uniqueC/Rt_uniqueC) designed for TaqMan was also tested by SYBR Green qPCR, which showed similar results (**Figure S1**). The specificity of this chromosomal unique gene was also tested using cDNA templates, and the cDNA sample from strain GH1-13 showed specific amplification compared to the sample from strain *B*. *velezensis* JC-14 (**Figure S2**). The specificity of the unique gene from plasmid DNA in GH1-13 was also assayed using SYBR Green qPCR analysis. The results indicated that this gene can be specifically amplified in strain GH1-13 using either DNA or cDNA templates when compared to strain JC-14 (*B*. *velezensis*). Meanwhile, the amplification of DNA template from GH1-13 showed 4.68 Ct values, on average lower than using the cDNA template (**Figure S3**). The qPCR assay detected the chromosomal unique gene of GH1-13 and showed high sensitivity amplifying 1 ng to 10 fg of target DNA by SYBR Green (**Figure S4A**) and TaqMan (**Figure S4B**) analyses. The linear relationships between serial diluted genomic DNA concentrations (log transformed) and Ct values were y = –3.24x + 39.95 (R^2^ = 0.98) in SYBR Green qPCR analysis and y = –2.94x + 38.62 (R^2^ = 0.98) in TaqMan qPCR analysis. The amplification efficiency was 103.53% in SYBR Green qPCR and 118.84% in Taqman qPCR.

### 3.7 Detection of GH1-13 from creeping bentgrass rhizosphere in the pots

**Figure 7:**
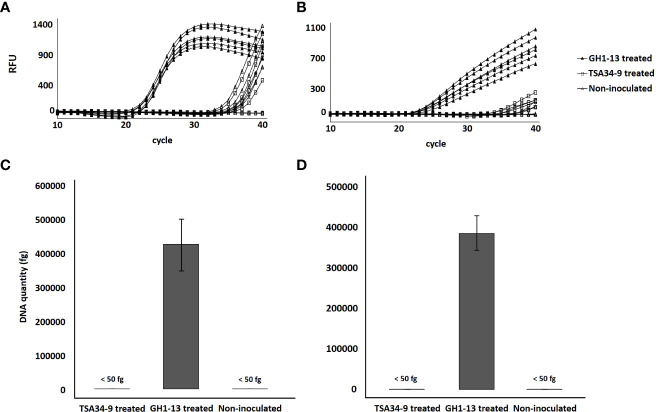
Amplification of chromosomal unique gene region of *Bacillus velezensis* GH1-13 from DNA templates of *B. velezensis* GH1-13 treated, *B. velezensis* TSA34-9 treated, and non-inoculated soil samples (500 mg) by SYBR Green qPCR analysis using a primer pair F_uniqueC and R_uniqueC **(A)** and TaqMan qPCR analysis using a primer pair Ft_uniqueC and Rt_uniqueC **(B)**. DNA quantity of chromosomal unique gene region of *B. velezensis* GH1-13 detected from *B. velezensis* GH1-13 treated, *B. velezensis* TSA34-9 treated and non-inoculated soil samples (500 mg) by SYBR Green qPCR analysis using a primer pair F_uniqueC and R_uniqueC **(C)** and TaqMan qPCR analysis using a primer pair Ft_uniqueC and Rt_uniqueC **(D)**.

Of the two unique genes, the chromosomal unique gene was selected for specific detection of the strain GH1-13 from creeping bentgrass rhizosphere soil. Only GH1-13 treated rhizosphere soil showed specific amplification by SYBR Green (**Figure 7A**) and TaqMan (**Figure 7B**) qPCR assays with average Ct values were 21.82 and 22.04, respectively. The average Ct values of TSA34-9 treated and non-treated samples all exceeded 35. The average DNA quantities detected in GH1-13 treated rhizosphere soil samples were 427,682 fg/µl and 387,165 fg/µl in SYBR Green and TaqMan qPCR analysis, respectively. For TSA34-9 treated and non-treated samples, the DNA quantities detected were all less than 50 fg/µl (**Figures 7C, D**).

### 3.8 Detection of GH1-13 from creeping bentgrass rhizosphere in the field

**Figure 8:**
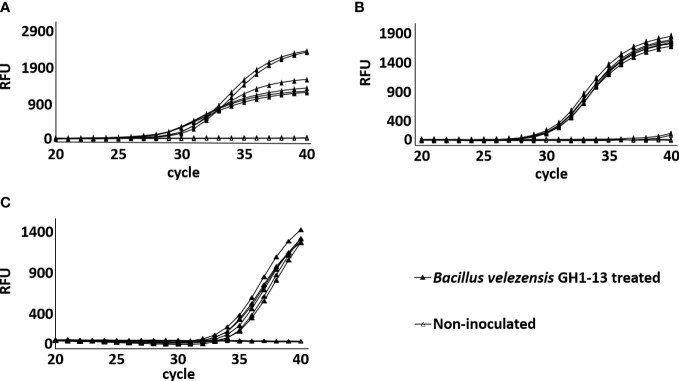
Amplification of chromosomal unique gene region of *Bacillus velezensis* GH1-13 by SYBR Green qPCR analysis from DNA templates of *B. velezensis* GH1-13 treated and non-inoculated soil samples (500 mg) collected in the field after treatment for 3 h **(A)**, 6 months **(B)** and 9 months **(C)**.

To verify the detection method in the field condition, GH1-13 was treated in creeping bentgrass field and detection was conducted using soil samples after 3 h, 6 months, and 9 months of treatment. The chromosomal unique gene region of GH1-13 was successfully amplified by SYBR qPCR in all the three post-treatment samples, while the samples from the control plots remained no amplification (**Figure 8**). The amplification curves of GH1-13 from long-term span samples (3h, 6 months, 9 months after treatment) suggested that the bacterial strain lives stably in the rhizosphere and can survive in the winter condition.

### 3.9 Field efficacy of GH1-13 for management of brown patch

The application of GH1-13, fungicide or the combination of GH1-13 and fungicide significantly reduced the brown patch disease in the field assays in 2021 and 2022. The disease severity in the plots treated with *R. solani* was significantly higher (78.79 ± 3.03%) than that of the fungicide azoxystrobin 100% (6.06 ± 6.06%) and GH1-13 (9.09 ± 5.25%) treatment along with the pathogen in 2021. The control efficacy of azoxystrobin, GH1-13, and their combined application was not significantly different. In 2022, the disease severity in the plots treated with *R. solani* also was significantly higher (51.52 ± 8.02%) than that of the fungicide azoxystrobin 100% (9.09 ± 5.25%) and GH1-13 (12.12 ± 3.03%) treatment along with the pathogen. The treatment of azoxystrobin (100%) and the combination of GH1-13 and azoxystrobin (50%) showed highest control efficacy (**Table 2**).

**Table 2:** Year wise effect of GH1-13 on the disease severity (DS) and control efficacy (CE) against brown patch disease caused by *Rhizoctonia solani* in the field assay.

Treatment	2021		2022	
	DS^*^ (%)	CE^*^ (%)	DS (%)	CE (%)
*R. solani*	78.79 ± 3.03 a	–	51.52 ± 8.02 a	–
*R. solani +* Azoxystrobin 50%	21.21 ± 10.93 b	73.08 ± 14.00 a	21.21 ± 3.03 b	58.83 ± 5.58 a
*R. solani* + Azoxystrobin100%	6.06 ± 6.06 b	92.31 ± 8.00 a	9.09 ± 5.25 b	82.35 ± 10.19 a
*R. solani* + GH1-13	9.09 ± 5.25 b	88.46 ± 6.66 a	12.12 ± 3.03 b	76.47 ± 5.88 a
*R. solani +* GH1-13 *+* Azoxystrobin 50%	15.15 ± 3.03 b	80.77 ± 3.85 a	9.09 ± 5.25 b	82.35 ± 10.19 a

^*^DS and CE indicates disease severity and control efficacy, respectively. Data of disease severity and control efficacy used in the table are the mean ± SE. Different lowercase letters after data indicate significant differences among treatments (p< 0.05). Values with the same letters are not significantly different.

## 4 Discussion

Due to increased fungicide-resistant fungal populations and environmental issues stemming from heavy applications of fungicides (Lamichhane et al., 2017), screening and discovering biocontrol agents have been widely pursued to manage turf diseases (Guerrero et al., 2008; Coelho et al., 2021). In this study, we found that *B. velezensis* strain GH1-13 can be a promising biological agent to control brown patch and showed that the application of the combination of biocontrol agent and fungicide was an alternative way to control the disease using reduced amounts of fungicide. In addition, using pan- genome analysis of genomes of GH1-13 and other *B. velezensis* strains and *Bacillus* species, unique genes in the chromosome and plasmid of GH1-13 were found and targeted for strain-specific detection. Two unique genes of GH1-13 were specifically detected using DNA and cDNA samples by SYBR Green and TaqMan qPCR analyses and strain-specific detection was successfully developed using DNA samples from creeping bentgrass rhizosphere soil and overwintered soil.

The strain GH1-13 showed a strong antifungal activity against fungal and oomycete species causing diseases of turfgrasses. Results of dual culture assay indicated that growth of *Rhizoctonia* species causing brown patch, large patch, and yellow patch was more inhibited by GH1-13 than growth of *C. jacksonii* and *P. ultimum*. This bacterial strain might provide more specific antagonistic activity against basidiomycete *Rhizoctonia* species than against ascomycete fungus and oomycete species. Dead cells of *R*. *solani* during its interaction with *B*. *velezensis* GH1-13 were microscopically observed using Evans blue staining. Also, observation of abnormal hyphae structures displaying roundish cells in the terminal hyphae may imply cessation of the hyphae extension. *Bacillus* species have been recognized as biological agents to control plant pathogens through various strategies, such as employing hydrolytic enzymes to inhibit the growth of pathogens and secreting antifungal compounds or lipopeptides (Khan et al., 2018). The genome of GH1-13 contains the biosynthetic gene clusters of antimicrobial compounds (e.g. amylocyclicin, bacilysin, fengycin, surfactin, and iturin) (Kim et al., 2017) and the lipopeptide compound, surfactin, was isolated from GH1-13 and inhibited the growth of fungal mycelia (Park et al., 2019). In addition to previous studies, our results of mycelial growth inhibition and even mycelial death by GH1-13 indicate that the bacterium could potentially act as a fungicidal agent.

Combining biological, chemical, and cultural approaches is often additive and sometimes synergistic, leading to more effective and reliable disease control. In managing soil-borne diseases of several crops, an integrated approach involving microbes and fungicides was found highly effective (Akhter et al., 2015). Akhter et al. (2015) applied combinations of *Trichoderma harzianum*, mustard oilcake, and fungicide to soil and seedlings to control *R. solani* on peas (*Pisum sativum* L.). This appeared to be significantly superior in reducing seedling mortality and improving seed yield in comparison to any single applications. In the current study, the strain GH1-13 was compatible with the commercial fungicides, azoxystrobin, and the combined application of GH1-13 and azoxystrobin effectively controlled brown patch. This combined application reduced 50% of the applied fungicide amount and showed control efficacy similar to application 100% of azoxystrobin in the pot and field assays. Interestingly, a significantly increased number of bacterial colonies were observed when the GH1-13 grew on 20 μg ml^-1^ of fluxapyroxad amended medium. This phenomenon might be due to hormetic effects. Hormesis is defined as a dose-response phenomenon characterized by low-dose stimulation and high-dose inhibition and represents an overcompensation for mild environmental stress (Kouda and Iki, 2010; Martel et al., 2019). Combining a small amount of fungicide with the biocontrol agent could offer a new disease management strategy to increase the number of biocontrol agents and reduce the amounts of fungicide.

With the rapid development of high-throughput sequencing technologies, more and more microbial genomes are available for large-scale comparative genomic analysis. With publicly available resources, it is possible to accomplish strain-specific detection for different purposes (Hernández et al., 2020). The concept of pan-genome was coined by Tettelin et al. in 2005, as an early example of mathematical modeling applied to biological big data. Such a data-driven approach for multi-genome study provides a platform to estimate the genomic diversity from a dataset, as well as determining the core, assessor, and unique genes of a species (strain), which is meaningful for understanding genomic dynamics, microbial population structure and other features. Many pan-genome programs have been designed and published, which suffer from one or more limitations and await improvement (Chaudhari et al., 2016). BPGA (Bacterial Pan Genome Analysis tool) is a newly developed ultra-fast and efficient computational pipeline with the functions of various downstream analyses (Chaudhari et al., 2016). This study employed the BPGA program to obtain strain specific genes (singletons) from GH1-13 among 364 genomes of *Bacillus* spp. Finding the two unique genes of GH1-13 served as an important basis for the subsequent detection of this specific strain.

Quantitative PCR assay is a powerful technique and has been well-established for the detection and quantification of different microbes, including pathogen diagnostics or biocontrol agent tracking (Hernández et al., 2020; Roth et al., 2020; Wang et al., 2020), but the method has been mostly limited to detection at the species level. In this study, SYBR Green and TaqMan qPCR analyses of unique genes in the chromosome and plasmid of *B. velezensis* strain GH1-13 showed high specificity for the strain GH1-13 compared with other strains of *B*. *velezensis* and other bacterial and fungal strains. Comparing these two methods, the TaqMan qPCR method displayed higher specificity (**Figure 6**), while the SYBR Green qPCR method can be a simpler and more economical option in detection and diagnostics. The previous study of Roth et al. (Roth et al., 2020) reported a diagnostic qPCR assay to detect *Fusarium brasiliense* and the limit of detection was determined to be 100 fg target DNA or a Ct ≥ 31. In this study, the amplification curves of other samples with high Ct values (over 35) in specificity assays or detection in soil might reflect non-specific amplification. The successful detection of strain GH1-13 in rhizosphere of creeping bentgrass in pots and in the field demonstrated the robustness and reliability of this detection method. Undoubtedly, efficient colonization of the rhizosphere by biocontrol agents is essential to control soil-borne diseases (Niu et al., 2020). The survival of GH1-13 in overwintered soil potentially indicates its stable colonization and role as a biocontrol agent and plant growth promoter. In addition, the detection method developed in this study also can be further applied on the study of community dynamics of other control agents. In a qPCR specificity assay using cDNA templates, the specific amplification of strain GH1-13 suggests that cDNA can be an ideal material for the determination of cell viability of the biocontrol agent, which cannot be achieved by DNA templates.

To manage turfgrass diseases by biological agents, organisms such as *B. licheniformis, B. subtilis, Gliocladium catenulatum*, *Pseudomona aureofaciens*, and *T. harzianum* have been applied to golf courses (https://extension.missouri.edu/publications/ipm1029?p=1). This study proposes that *B. velezensis* GH1-13 can be a new biological agent for brown patch management and combining a fungicide with GH1-13 can efficiently reduce diseases as well as the proportions of fungicide application. The pipelines of bacterial strain-specific detection methods using both pan- genome and RT-qPCR analysis developed in this study can prove useful for subsequent studies of biocontrol agents for detecting and investigating colonization and dynamics of these agents.

## Data availability statement

The original contributions presented in the study are included in the article/**Supplementary Material**. Further inquiries can be directed to the corresponding author.

## Author contributions

HS conceptualized, designed, edited, and managed funding to conduct the research. GL, HC, HL, and Y-HH performed most of the experimental work and data analysis. NP, GH, HK, PK, S-IS, and JS conducted some parts of the experiment. GL, HC, HL, Y-HH, NP, and HS contributed to the writing of the manuscript. All authors contributed to the article and approved the submitted version.
