# Supplementary material for: Biocontrol of the causal brown patch pathogen Rhizoctonia solani by Bacillus velezensis GH1-13 and development of a bacterial strain specific detection method
Source: Front Plant Sci. 2023 Jan 9;13:1091030. doi: 10.3389/fpls.2022.1091030 (PMC9868939; doi:10.3389/fpls.2022.1091030)
Supplement: Supplementary file 1 [file DataSheet_1.pdf]

Supplementary Material

Supplementary Figures

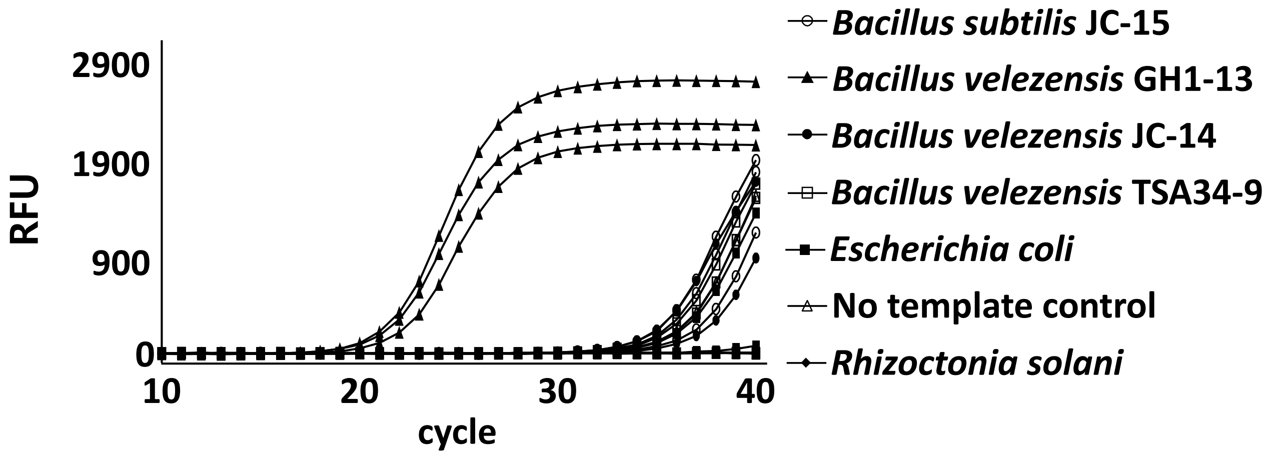

Supplementary Figure S1. Amplification of 0.1 ng initial DNA of *Bacillus velezensis* GH1-13, *B. velezensis* JC-14, *B. velezensis* TSA34-9, *B. subtilis* JC-15, *Escherichia coli*, *Rhizoctonia solani* and no template control by SYBR qPCR analysis using a primer pair Ft_uniqueC/Rt_uniqueC.

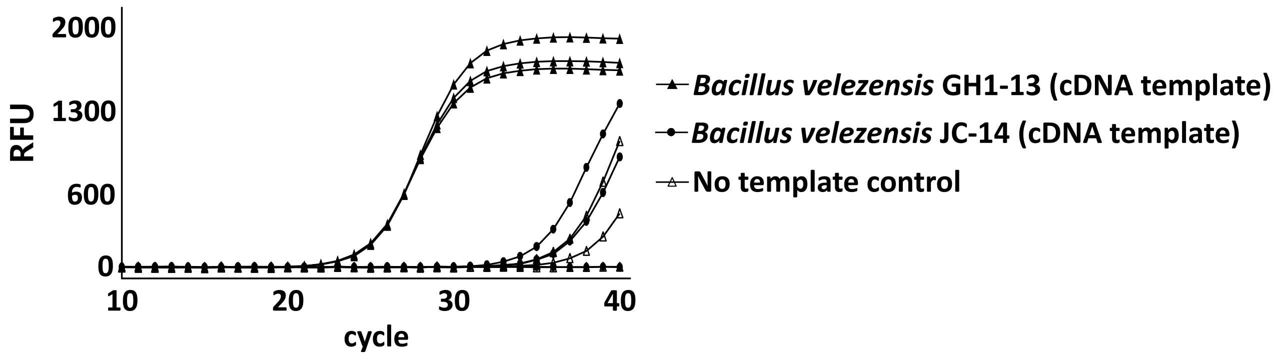

Supplementary Figure S2. Amplification of chromosomal unique gene region of *Bacillus velezensis* GH1-13 from cDNA templates of *B. velezensis* GH1-13, *B. velezensis* JC-14 and no template control by SYBR qPCR analysis using a primer pair F_uniqueC and R_uniqueC.

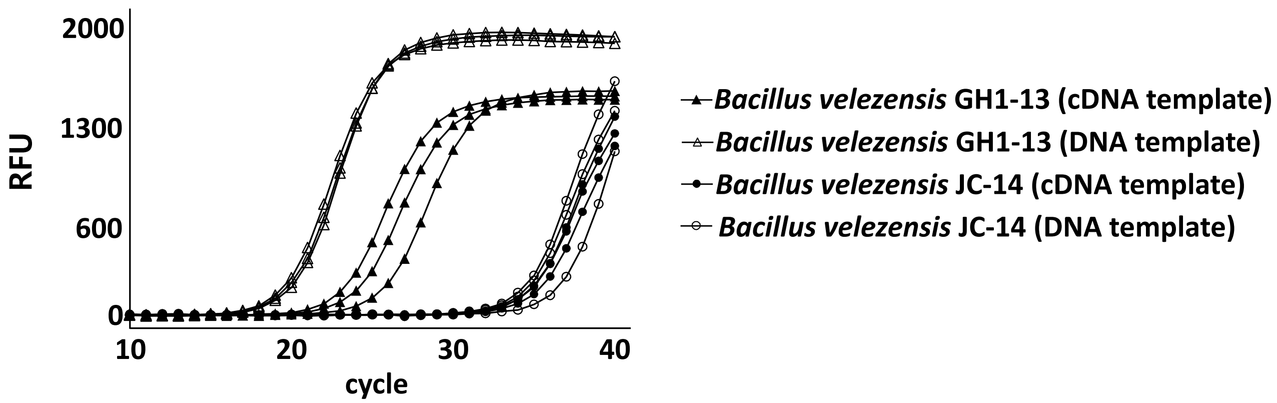

Supplementary Figure S3. Amplification of plasmid unique gene region of *Bacillus velezensis* GH1-13 from both DNA and cDNA templates of *B. velezensis* GH1-13 and *B. velezensis* JC-14 by SYBR qPCR analysis using a primer pair F_uniqueP and R_uniqueP.

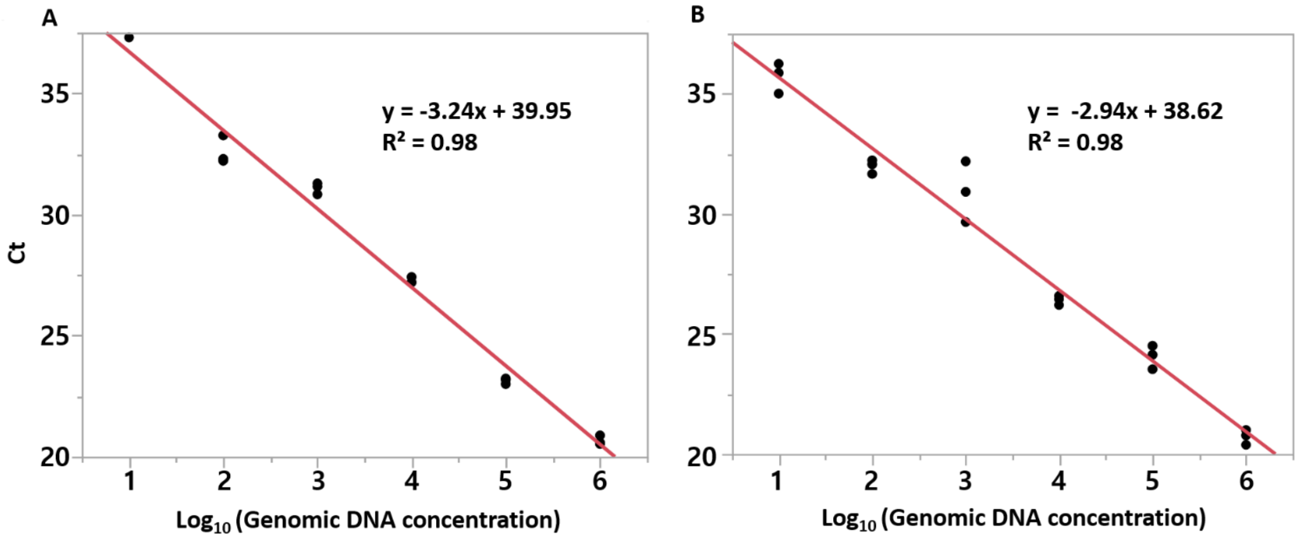

Supplementary Figure S4. Calibration curves of SYBR Green (A) and TaqMan (B) qPCR analyses quantifications plotted with serially diluted *B. velezensis* GH1-13 genomic DNA (log transformed) in femtograms against cycle threshold values using primer pairs (F_uniqueC and R_uniqueC for SYBR Green qPCR analysis and Ft_uniqueC and Rt_uniqueC for TaqMan qPCR analysis) targeting the chromosomal unique gene region.
